# Instrumental Monitoring of a Slow-Moving Landslide in Piedmont (Northwest Italy) for the Definition of Rainfall Thresholds

**DOI:** 10.3390/s24113327

**Published:** 2024-05-23

**Authors:** Mauro Bonasera, Battista Taboni, Chiara Caselle, Fiorella Acquaotta, Giandomenico Fubelli, Luciano Masciocco, Sabrina Maria Rita Bonetto, Anna Maria Ferrero, Gessica Umili

**Affiliations:** 1 Geological Survey of Italy, Italian Institute for Enviromental Protection and Research—ISPRA, Via Vitaliano Brancati 48, 00144 Rome, Italy; mauro.bonasera@isprambiente.it; 2 Department of Earth Sciences, University of Turin, Via Valperga Caluso 35, 10125 Turin, Italy; battista.taboni@unito.it (B.T.); chiara.caselle@unito.it (C.C.); fiorella.acquaotta@unito.it (F.A.); giandomenico.fubelli@unito.it (G.F.); luciano.masciocco@unito.it (L.M.); sabrina.bonetto@unito.it (S.M.R.B.); gessica.umili@unito.it (G.U.)

**Keywords:** landslides, geological model, monitoring, inclinometer, rain thresholds, InSAR

## Abstract

The prediction and prevention of landslide hazard is a challenging topic involving the assessment and quantitative evaluation of several elements: geological and geomorphological setting, rainfalls, and ground motion. This paper presents the multi-approach investigation of the Nevissano landslide (Asti Province, Piedmont, NW Italy). It shows a continuous and slow movement, alongside few paroxysmal events, the last recorded in 2016. The geological and geomorphological models were defined through a field survey. An inventory of the landslide’s movements and rainfall records in the period 2000–2016 was performed, respectively, through archive investigations and the application of “Moving Sum of Daily Rainfall” method, allowing for the definition of rain thresholds for the landslide activation (105 mm and 193 mm, respectively, in 3 and 30 days prior to the event). The displacements over the last 8 years (2016–2023) were monitored through an innovative in-continuum monitoring inclinometric system and Earth Observation (EO) data (i.e., relying on Interferometric Synthetic Aperture Radar, or InSAR data): it gave the opportunity to validate the rainfall thresholds previously defined. This study aims to provide information to public authorities for the appropriate management of the site. Moreover, the proposed workflow could be adopted as a guideline for investigating similar situations.

## 1. Introduction

Landslides represent one of the most serious hazards in many areas of the world. Their activation can be related to several factors, including rainfall, earthquakes, snowstorms and human activities (e.g., [1,2,3,4,5,6]). In large portions of the Italian territory, landslide susceptibility is high to very high due to the combination of morphological, geological and climatic factors, and to the additional effect of human activity (e.g., [7,8,9,10]).

Among others, rainfall is known to be one of the most common triggering factors for landslide activation. Rain infiltration in the slope sediments causes an increase in the pore pressure and a decrease in the soil suction, up to the critical values that trigger the landslide activation. The capability of a rainfall event to trigger the landslides’ initiation is constrained by the combination of different elements, including the intensity and the duration of the pluviometric event itself, the geological, geomorphological, hydrogeological and geotechnical characteristics of the slope and soil moisture [11,12]. Significant efforts have been made by the scientific community to analyse the relationship between the amount of rainfall and landslide initiation, aiming to identify the critical rainfall thresholds for the triggering of shallow landslides [13,14,15]. Due to the variability of the involved parameters, the amount of rainfall required to trigger a landslide can differ even for adjacent slopes. This means that, in addition to general threshold values at a regional level, the identification of site-specific thresholds is often useful.

Following the definition by [15], in the present study, we focused on the identification of the “minimum triggering rainfall threshold”, i.e., the minimum value of precipitation below which the process is never triggered. More specifically, we used the “Moving Sum of Daily Rainfall” empirical approach (previously proposed by [16,17]), based on the comparison between different series of cumulative daily rainfall data and the behaviour of the landslide. However, rainfall thresholds obtained with empirical approaches such as the “Moving Sum” method need to be tested, calibrated, and validated through rigorous instrumental measurements [18]. In this regard, in recent years, scientific and industrial research has provided innovative landslide monitoring systems able to achieve high sampling frequencies with automatic and remotely controlled procedures [19,20,21,22,23,24].

In addition to contact sensors for the quantification of displacement, in recent decades, the utility of Interferometric SAR (InSAR) to identify, map and monitor landslides has been demonstrated [25] and, as a consequence, InSAR has become consolidated in the field of geotechnics as a tool for the study of landslides. It is largely used for the study of landslide’s reactivation [26], for the characterization of precursors and triggering factors [27], for the comparison between landslide behaviour and rainfall characteristics [28] and for landslide inventory purposes [29], among others (for a complete review, see [30]).

In this study, we chose the Nevissano landslide (Castelnuovo Don Bosco, Piedmont, NW Italy) as a test site to cross-validate the “Moving Sum” empirical method and the instrumental measurements acquired through an innovative monitoring system (Modular Underground Monitoring System (MUMS) technology, produced by ASE S.r.l., [31]). Over recent decades, the Nevissano landslide recorded several re-activations spread over time (i.e., landslide paroxysmal events), the last of which was in 2016. Hence, we used the data about rainfalls and paroxysmal events over a time period of 17 years (between 2000 and 2017) to empirically define the most suitable minimum rainfall thresholds, based on the “Moving Sum” approach.

In 2017, the MUMS inclinometric monitoring system was installed on site. Thus, we compared the MUMS time-displacement data series and the derived time-velocity and time-acceleration data series with the rainfall trends over the 2017–2023 period, to validate the proposed thresholds. Lastly, the inclinometric data provided by the MUMS sensor was compared with available Interferometric Synthetic Aperture Radar (InSAR) data provided by [32,33,34]. This was performed in order to enlarge the dataset of ground motion data, also introducing a different source of data not related to the in-continuum acquisition of the MUMS inclinometer. Even if the absence of paroxysmal events during the monitoring period did not allow for a complete validation of the thresholds, the comparison provides useful insights for the interpretation of rainfall series in similar contexts. Moreover, we highlighted some possible criticalities in the use of high-frequency inclinometric data, particularly if related to misuse of the instrumentation.

## 2. Geological Setting

### 2.1. Regional Geology

The study area is located in Piedmont (NW Italy), near a location called Nevissano, in Castelnuovo Don Bosco municipality (Province of Asti). The area is part of the Turin Hill, i.e., the north-western sector of Tertiary Piedmont Basin (TPB), a sedimentary sequence located on the inner side of SW Alps. The TPB is filled with Upper Eocene to Messinian sediments, prevalently of marine origin, that stratigraphically overlies a complex tectonic wedge of Alpine, Ligurian and Adria basement units juxtaposed in response to the collision between the Europe and Adria plates [35]. The Cenozoic sediments are presently exposed in the southern (Langhe, Alto Monferrato and Borbera Grue domains) and the northern (Torino Hill–Monferrato arc) sectors of the TPB. The relationships between the two outcropping sectors are masked by Pliocene-to-Holocene deposits of the Savigliano and Alessandria basins, but are well-imaged by seismic profiles [36].

### 2.2. Local Geology and Geomorphology

**Figure 1:**
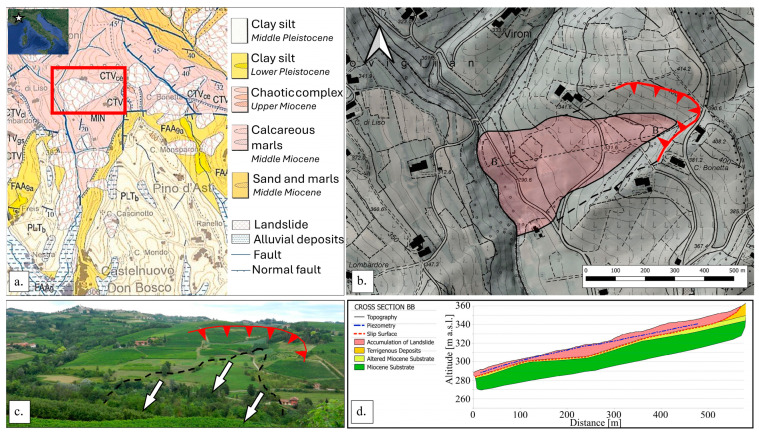
(**a**) Geological map of the study area [38]. The landslide body is highlighted by the red rectangle; (**b**) accumulation body and main scarp of the Nevissano landslide; (**c**) landscape image of the Nevissano landslide with identification of the main scarp (rad line), of the accumulation body (black dashed line) and of the main sliding direction (white arrows); (**d**) schematic cross section from [39].

The test site is included in both the Geological Map of Italy at the scale of 1:100,000 (56th and 57th sheets, Torino and Vercelli) and the Geological Map of Italy at the scale 1:50,000 (156th sheet—Torino Est, Figure 1a), alongside its description in the relative illustrative notes [37,38]. The area predominantly consists of Miocene marly sediments belonging to the so-called “Marne di Mincengo” unit.

The geomorphological features of the area are mainly controlled by rill erosion and the evolution of the drainage network. Slope alteration and mass solid transport are associated with the deepening of stream channels due to the erosive action of water. The drainage pattern is dendritic, with several short streams, which converge in a few larger watercourses.

The municipality of Castelnuovo Don Bosco has a medium-to-low attitude to slope instability processes. The hilly portion of the area hosts several ancient or active shallow landslides that involve eluvial-colluvial deposits. They usually involve small volumes, and can affect even moderately steep sectors, not exceeding a few tens of metres in length. These shallow landslides are related to abundant rainfalls, which completely soak the superficial layer of the ground, activating the sliding. These kinds of events could coexist with processes with different kinematics and dimensions (e.g., rotational slides that may evolve in debris flows in the lower portion of the involved volume).

Landslide susceptibility in the area is also enhanced by two additional factors: very high slope steepness and soil consolidation reduction related to agriculture.

### 2.3. The Nevissano Landslide

The Nevissano landslide (Figure 1b,c) has an approximate extension of 0.2 km^2^, with a main body of about 600 m in length and a maximum width at the base of the slope of 350 m. The apparent movement is from NNE towards SSW. However, due to the main south-oriented dip direction of geological strata, the landslide movement is supposed to turn southward in depth. Toward ENE, the landslide sector is limited by a large steep scarp, bordering the landslide body upslope and on its side. These steep sectors of the slope separate the landslide area from external elevated areas, which host two farmhouses.

The landslide body shows several undulations and counterslopes. At the bottom, the actual deposit forces the path of the Nevissano River abnormally close to the cliffs on the opposite bank. The road that crosses the landslide sector (SC33 Nevissano) is the clearest marker of landslide movements. In detail, the path of the road highlights the oldest movements, being translated downstream, and suggests recent reactivations of the south-eastern part of the landslide. The whole phenomenon is active, but its movements presumably do not involve all the wide area simultaneously with the same intensity. Reactivations (or, at least, the most detectable reactivations) seem to be concentrated in the area of the road itself. In recent decades, reported movements of the road have been mostly slow, but constant and recurrent, especially in the northernmost section of the track. Most of them occurred during rainy seasons.

A detailed geological and geotechnical investigation performed in 2013 by [39] allowed for the definition of the geological sequence of the slope, summarized in the profile in Figure 1d. Such a profile describes a hypothetical sliding surface (red dashed line in Figure 1d) corresponding to a sudden increase in geotechnical strength properties observed in borehole logs. The proposed surface is sub-parallel to the slope and lies at a depth ranging between 4–5 m and 7–8 m along the road path; this value increases toward the centre of the landslide body up to a maximum of about 12–13 m. The sliding surface becomes vertical, reaching the ground surface toward ENE, coherently with the location of the crown scarp. The geotechnical analyses provided by [39] also included the measurement of groundwater level. As reported in Figure 1d (dashed blue line), at the time of measurement, the groundwater level was identified at a maximum depth of 3–5 m in the upper portion of the landslide body, at 2–4 m in the central portion, and at a minimum of 1.5–2 m at the foot of the landslide body, confirming the importance of water in its re-activations.

## 3. Materials and Methods

### 3.1. Definition of Rain Thresholds

For the purpose of defining rain thresholds for the activation of paroxysmal events of the Nevissano landslide, we employed the “Moving Sum of Daily Rainfall” empirical approach [16,17]. From the agro-meteoric network of the Piedmont Region, we collected the daily rain data of Castelnuovo Don Bosco covering the period between 2000 and 2017 and elaborated them to define different series of cumulative daily rainfalls for increasing intervals of time. More specifically, we defined six numerical series for time intervals of 3, 7, 15, 30, 60, and 120 days, respectively. Then, we estimated the dates of the main paroxysmal events of the Nevissano landslide by considering information provided by municipal and regional offices, local chronicles records, municipal ordinances that closed the SC33 Nevissano Road, and scientific literature [40]. Based on these two independently obtained data series, we empirically evaluated the most representative minimum rain thresholds for the activation of the Nevissano landslide. In correspondence with each of the identified paroxysms’ dates, we studied the value and the shape of the peaks in a cumulative rain-vs-time graph, looking for the values that could be effective thresholds for all the considered events and focusing on the well-defined peaks with a high gradient. As a further step, we checked the total number of threshold exceedances in the whole dataset to verify the performance of the chosen thresholds. For excellent performance, the number of thresholds’ exceedances should be as close as possible to the number of landslide events.

### 3.2. Monitoring of the Landslide

#### 3.2.1. Monitoring System

After the paroxysmal event of 2016, a remotely controlled monitoring system (namely, MUMS system, by ASE S.r.l., [31]) was installed on-site, in correspondence with the SC33 Nevissano Road. The system consists of an array of 24 nodes, installed in a borehole at depths ranging between 0.25 m and 11.75 m and anchored to the ground at a depth of 12 m. Each node is equipped with three sensors: (i) a 3D Micro Electro-Mechanical Systems (MEMS), (ii) a thermometer, and (iii) a magnetometer. Thanks to the combination of the independent measurements acquired by these three sensors, the system provides spatially oriented displacement data for each node (i.e., for each considered depth): the 3D MEMS, calibrated on the basis of the temperature recorded from the internal thermometer, provides the actual displacement value, while the magnetometer provides its spatial orientation with respect to the N. The system records data at time intervals of 1 h, and automatically transmits them to a mainframe server. Additional technical specifications about data acquisition, recording and remote access are available at [31].

#### 3.2.2. Data Elaboration

Analogously to the procedure commonly used for traditional inclinometers, we elaborated the MUMS displacement data to retrieve the cumulated displacements (i.e., the sum of the local displacements measured in all the nodes for a considered depth interval). Using the N-S and E-W components of the displacement, we separately calculated the cumulated displacements along these two main directions. Then, by combining the N-S and the E-W components, we calculated the actual value and orientation of the displacement for each acquisition at each node. We then differentiated the cumulated displacement with respect to time, in order to obtain the velocity and the acceleration for the entire dataset. More specifically, we linearly fitted the displacement and, for the second-order differentiation, the velocity over time intervals of 30 days, in order to minimize the influence of high-frequency displacement fluctuations.

This approach implies some issues that, if not correctly addressed, may affect the accuracy and reliability of the resulting data, especially if the high-frequency-recorded dataset is elaborated through automatic or semi-automatic algorithms. In detail, these issues are related to: (i) the missing registration of one acquisition; (ii) the overestimation of displacement data at the basis of the nodes’ array; and (iii) the existence of magnetic anomalies.

In case of missing registration of one acquisition, the data series will simply include the absence of one datum in correspondence with one of the nodes. However, due to the method used to retrieve the total (cumulated) displacement, the absence of one datum also affects the total displacements in the shallower nodes, even if those nodes correctly registered the local displacement value.

On the other hand, in case of overestimation of displacement data at the basis of the nodes’ array, the overestimation will affect all the nodes along the considered depth interval. In our specific case study, the first (i.e., the deepest) node is posed at only 25 cm from the anchoring (placed at 12 m of depth). Hence, small noisy inclinations of the sensor may be registered as displacements and, in turn, affect the cumulated displacement recordings of the entire column.

Lastly, due to the fact that displacements and their orientation are separately acquired from two different sensors (3D MEMS and magnetometer), if the magnetometer registers some anomalous data (e.g., due to external magnetic interferences), this again affects the entire column: the cumulated displacement will be the result of the sum of components oriented in different directions and, hence, will show anomalous trends (e.g., apparent backward displacements).

#### 3.2.3. Calibration after Shutdown

Due to both an administrative issue and the quarantine established from the beginning of 2020 for the COVID-19 disease, we temporarily could not provide ordinary maintenance to the MUMS system, with the result that it went offline between November 2019 and June 2022. After this period, a calibration of the new measurements starting point was necessary in order to assure the reliability of the acquired data. For this purpose, we compared the inclinometric data with the interferometric European Ground Motion Service (EGMS) monitoring network [32,33,34], which provides discrete geospatial layers containing purely vertical [41] and purely east–west [42] displacement data of the ground for a network of target points spread over the entirety of Europe. Two time series are nowadays available, with a sampling interval of six days and covering the periods between 2015–2021 and 2018–2022, respectively. The EGMS exploits a space-based remote sensing technology, using Synthetic Aperture Radar Interferometry (InSAR) data derived from Sentinel-1 to detect and measure ground movements across Europe with millimetre precision.

### 3.3. Validation of Rain Thresholds

In order to check the reliability of the estimated rain thresholds, we computed six series of cumulative daily rain data covering the period between 2016 and 2023, comparing them with the quantitative displacement data obtained from the MUMS array and with the derived velocity and acceleration data.

## 4. Results

### 4.1. Definition of Rain Thresholds

Table 1 reports the six dates of paroxysmal activation of the Nevissano landslide identified over the period 2000–2016. For each of them, the correspondent peak values of cumulative rainfall for periods of 3, 7, 15, 30, 60 and 120 days are reported. Five out of the six events already show quite high values (between 100 and 150 mm) for the shortest period (3 days). The only exception is represented by the paroxysm of the 6th of March 2002, having a cumulative rain height of 40 mm for the 3-day series. For this event, however, a drastic increase in rain height is observed for the 30 days series. Since this event happened less than 30 days after the previous considered event (on the 15 February 2002), this second movement was likely caused by the occurrence of a new moderate pluviometric event on a slope with a still-high water content. Hence, for the event of the 6th of March, the cumulative rain amount for 30 days seems to be the most significant datum.

**Figure 2:**
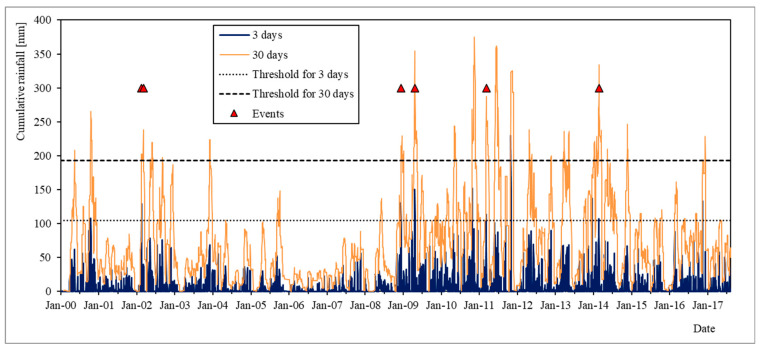
3-day and 30-day cumulative rainfall series and relative selected thresholds compared with the dates of the paroxysmal events of the landslide.

Based on this evidence, we set rain thresholds for the 3 and 30 day series at 105 mm (the minimum value, if the 6 March 2002 is neglected) and 193 (the global minimum value), respectively. These thresholds are graphically shown in Figure 2, together with the dates of the considered paroxysm events and the cumulative rainfall series for 3 and 30 days. The choice of the 3 and 30 days series is also compatible with the geological model of the landslide, since 3 rainy days are sufficient to saturate only the shallow layer, while 30 rainy days allow water to reach the sliding surface.

Table 2 reports data on the total number of rainfall events exceeding the two rain thresholds over the entire 2000–2017 period. As can be seen, the model performs very well, with a percentage of threshold-exceeding rainfall events without landslide activation of only 0.06%.

### 4.2. Monitoring of the Landslide

**Figure 3:**
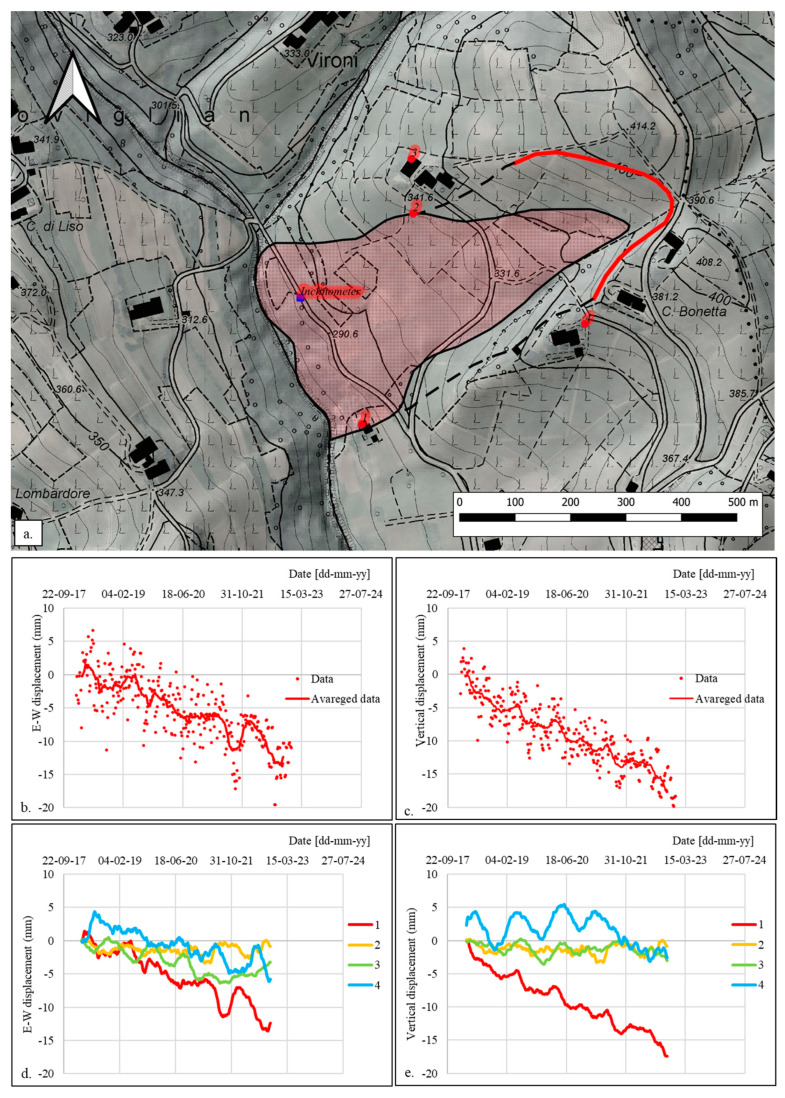
(**a**) Location of the MUMS inclinometer system and of the 4 selected target points of the EGMS network with respect to the position of the accumulation body of the Nevissano landslide; (**b**) E-W displacements at point 1 of the EGMS network; (**c**) vertical displacements at point 1 of the EGMS network; (**d**) E-W displacements at the four selected points; (**e**) vertical displacements at the four selected points.

Figure 3a shows the position of the installed MUMS monitoring system and the four selected target points of the EGMS InSAR network with respect to the Nevissano landslide. The EGMS InSAR data over the 2018–2022 period are summarized in Figure 3b–d. As shown in Figure 3b,c, the original data are quite noisy. For this reason, we averaged them over groups of 20 data, obtaining a well-defined displacement trend, both for E-W (Figure 3b) and vertical (Figure 3c) directions. Figure 3d,e report the (averaged) data of the four considered points. Points 2, 3, and 4 show a total amount of displacement within a range of ±5 mm both in E-W and vertical directions: thus, they can be considered stationary points located outside the landslide. On the other hand, point 1 registers a clearly defined downward displacement trend directed westward, describing negative values of vertical and E-W displacement.

**Figure 4:**
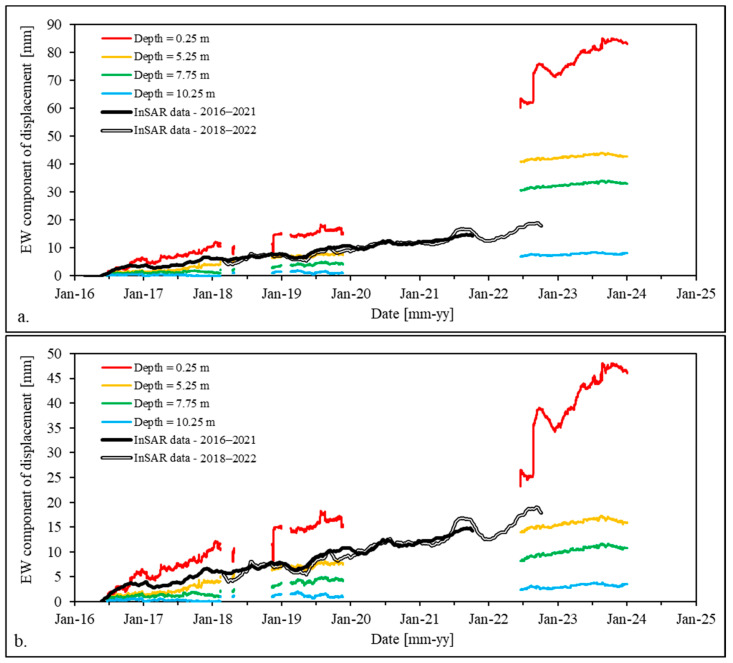
Comparison of E-W component of the displacement recorded by the MUMS system and at point 1 of the EGMS network before (**a**) and after (**b**) the post-shutdown calibration.

As shown in Figure 3a, point 1 of the EGMS InSAR network and the MUMS sensor array location fall in the same portion of the landslide (i.e., their distances from the head of the landslide and the crown main scarp are approximately the same). Hence, we compared the E-W components of the displacement recorded from the two systems (Figure 4a). Until the end of 2019, InSAR data show good agreement with MUMS data at a depth of 5.25 m (yellow curve). This is consistent, if a certain inhomogeneity of the movements of the shallower portion of the slide is assumed (e.g., faster displacements of shallow portions in the central part of the landslide—captured by the MUMS system—with respect to the peripheric position of point 1). This correspondence is lost after 2022, when the MUMS curves appear translated toward higher values of displacement. As previously discussed, after the shutdown of the instrument, the data had to be re-calibrated with respect to a known value of displacement. We consequently operated a downward translation of all the MUMS data from 2022 to 2024 based on the correspondence between the yellow curve (depth = 5.25 m) and the InSAR data. The results are shown in Figure 4b.

**Figure 5:**
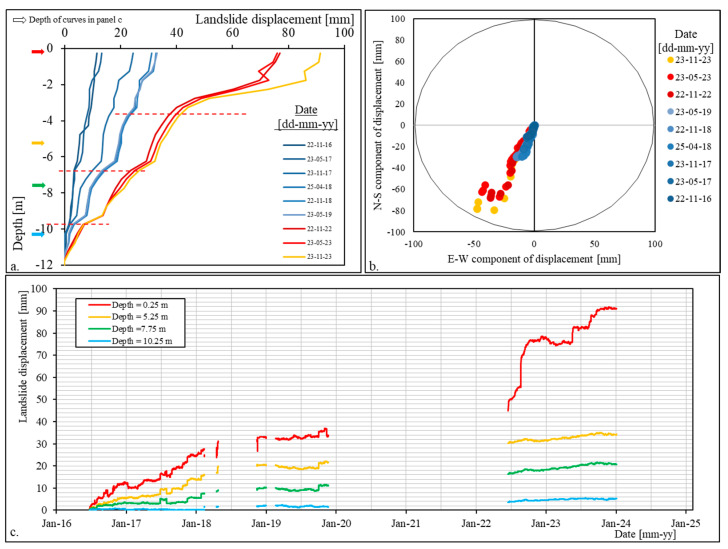
(**a**) Depth-displacement data for 9 dates spaced of 6 months. The red dashed lines refer to the depth of the identified sliding zones; (**b**) orientation of the displacements in the same dates of data reported in panel (**a**); (**c**) displacement-time curves for 4 selected nodes located at the depths graphically shown in panel (**a**) as coloured arrows.

A summary of the MUMS (cumulated) displacement data is presented in Figure 5. Figure 5a depicts nine series of displacement data as a function of depth, spaced every six months. Due to the absence of data between November 2019 and June 2022, the time interval between the blue (2019) and red–yellow (2022) curves is obviously referred to a time interval longer than 6 months.

The curves identify three main sliding surfaces or, rather, sliding zones (represented as red dashed lines in Figure 5a). These are localized as follows:

- Between 3 and 4 m of depth;
- Between 6 and 7 m of depth;
- Between 9 and 10 m of depth.

The shallower sliding surface (between 3 and 4 m of depth) has apparently been inactive since the beginning of the monitoring period, as displacement is recognisable only from November 2017. However, it appears to be the main element responsible for the recent evolution of the Nevissano landslide (from 2022 to the end of the period—red–yellow curves).

Figure 5b presents the orientation of the displacements recorded in the 24 nodes for the nine series of Figure 5a. The graph was obtained by plotting the E-W and the N-S components of the displacement on the x- and y-axes. As can be seen, the displacement data are quite homogeneously oriented toward SSW, consistently with field observations (see Section 2.3). In the most recent period (red–yellow series), the shallower points (i.e., with higher displacement values) show some dispersion, consistent with the high shallow displacements and apparent downward movement direction shown in Figure 5a.

Figure 5c shows the displacement-vs.-time plot for four nodes at depths of 0.25, 5.25, 7.75, and 10.25 m, respectively. These depths (indicated in Figure 5a as coloured arrows on the left) were selected on the basis of the sliding surfaces identified in Figure 5a, in order to describe portions of the landslide body behaving coherently. As can be seen, Figure 5c describes higher displacements (up to 30 mm for the shallower node) until the beginning of 2018. Then, the trend seems to change, describing almost no displacement at all nodes up to the end of 2023; the only exception being the shallowest node, which, as already been identified by Figure 5a,b, registered high displacements in 2022–2023, consistent with the activation of the new shallow sliding surface at 3–4 m of depth.

### 4.3. Validation of Rain Thresholds

**Figure 6:**
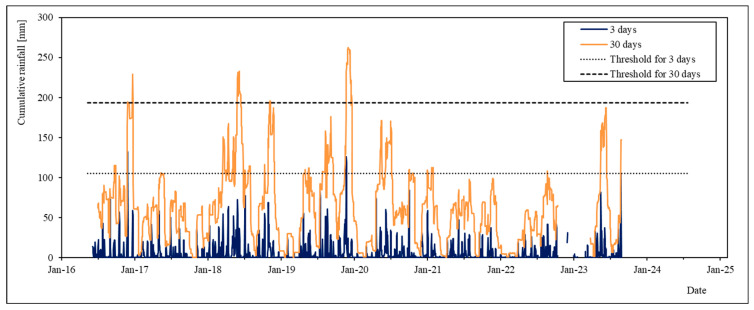
Cumulative rainfall series for 3 and 30 days for the period of 2016–2023.

Figure 6 reports the 3-day and 30-day cumulated rainfall data series for the 2016–2023 period. During this period, the 3-day threshold (dotted line) was overcome two times, while the 30-day threshold (dashed line) was overcome four times. Nevertheless, as previously said, the slope never registered paroxysmal events during the monitoring period.

**Figure 7:**
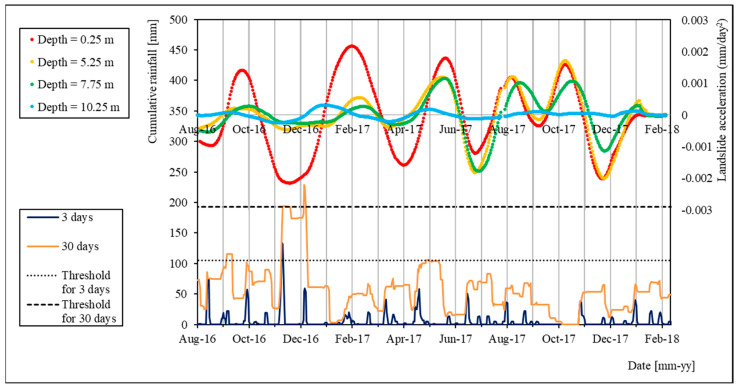
Comparison of landslide acceleration and cumulative rainfall series for the period of August 2016–February 2018.

**Figure 8:**
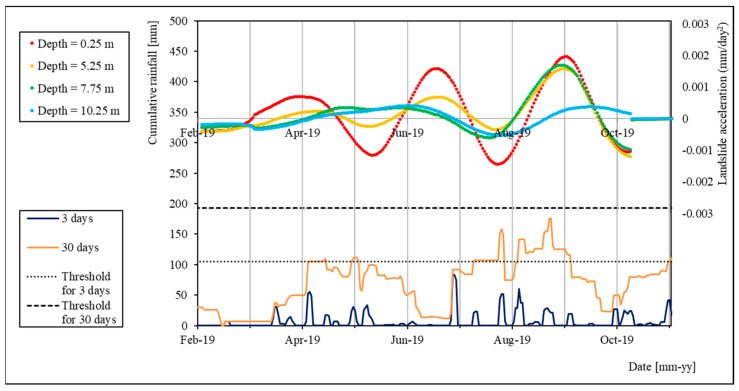
Comparison of landslide acceleration and cumulative rainfall series for the period of February–November 2019.

In Figure 7 and Figure 8, we propose a comparison between the cumulated rain series and the computed acceleration of the landslide for two time periods: (i) August 2016 to February 2018, and (ii) February 2019 to December 2019.

During the first period (Figure 7), the acceleration peaks generally show a similar trend to rain peaks. The main exception is represented by the acceleration peak in February 2017, which is shifted by approximately two months with respect to the rain peak (between November and December 2016, with overcoming of both 3-day and 30-day thresholds). This shifting of the slope movement with respect to rainfall could be explained by considering the influence of freezing of underground water and soil during winter on the increasing of the shear strength.

During the second period (Figure 8), the consistency between acceleration and rain trends is even clearer. As can be seen, the landslide presents three main acceleration peaks for the shallowest node (red curve). The first and second peaks correspond to peaks in 3-day curve of cumulative rain, while the third one shows good agreement with the 30-day curve of cumulative rain.

In the deeper nodes, the first peak is shifted by about one month, showing good agreement with the 30-day rain curve, consistently with the time required for the water to arrive at the deeper sliding surfaces.

In the last peak (September 2019), on the other hand, all the considered acceleration curves (except the 10.25 depth one—the light-blue curve) show the same trend, suggesting that the registered displacement happened along the deepest sliding surface (between 9 and 10 m of depth). This peak corresponds to a peak of the 30-day cumulated rainfall curve, with a maximum at 176 mm of rain: less than 20 mm lower than the considered threshold (193 mm).

## 5. Discussion and Conclusions

We reported a multi-approach investigation of the Nevissano landslide in Piedmont (NW Italy). The landslide behaviour is characterized by continuous millimetric displacements and periodical paroxysm re-activations, likely controlled by rainfall. We used the empirical “Moving Sum of Daily Rainfall” method to define rainfall thresholds for the activation of paroxysmal events of the landslide based on a data series over a time period between 2000 and 2017. In 2016, a high-frequency inclinometric monitoring system (MUMS) was installed on-site. Hence, we elaborated monitoring data over the time interval between 2016 and 2023 to obtain displacement, velocity and acceleration data series for a validation of the rain thresholds. Even if the absence of a paroxysmal event during the monitoring period prevented a complete validation of the thresholds, the comparison between rainfall trends and high-frequency displacement data confirms the relationship between the two physical quantities.

We used the acquired dataset to highlight some of the possible criticisms that may be encountered in the automatic elaboration of high-frequency inclinometric datasets. Since the total displacement at each node is calculated using a cumulative approach, the missing or noising registration of displacement or orientation data may affect the accuracy and reliability of the entire amount of data. Using non-supervised automatic elaboration approaches, similar errors may result in the misestimation of the actual landslide displacement.

The existence of biases associated with displacement datasets acquired by automatic inclinometers was already observed by other researchers. In particular, errors associated with (i) noise due to the acquisition system, (ii) systematic errors associated with damages to the acquisition system or to the probes, (iii) shifts caused by the removal of the in-place probe, and (iv) occasional errors due to unforeseen and unknown phenomena were observed [43].

Recently, Bordoni et al. [44] proposed a structured methodology for the correction of errors that may affect these kinds of measures. The approach is based on the idea that the displacement measured in a single day should never be larger than the standard deviation of the entire dataset. In addition, they also suggested to consider negative displacement, stressing the idea that also the measured direction of the movement acquired by the inclinometer can be used as an indicator of the measures reliability.

In the same study, the authors suggested that the experimental results can achieve the most significant impact at a large scale if datasets of displacement time-series acquired by automatic inclinometers are compared with climatic datasets (e.g., rainfalls or snowmelts), as was performed in the present study.

Eventually, our study identified that, in case of long periods of shutdown, the calibration of the sensors may be compromised; the first data recorded in the new data series may not correspond to the entity of displacement occurred during the shutdown. The comparison of inclinometric data with an independent measurement of landslide displacement (e.g., open source InSAR data) may be used to ensure that all data are correctly elaborated and interpreted, certifying the reliability and accuracy of the dataset.

## Figures and Tables

**Table 1:** Main paroxysmal event identified for the Nevissano landslide during the period 2022–2016 and the corresponding peak values of cumulative rainfalls for 3, 7, 15, 30, 60 and 120 days.

Paroxysmal Activation of the Monitored Landslide	Summary of the Cumulative Events					
Days of Events Recorded	3	7	15	30	60	120
	days	days	days	days	days	days
15 February 2022	120.20	120.20	183.20	193.40	193.40	253.60
6 March 2002	40.00	47.20	47.20	238.80	249.60	278.60
16 December 2008	130.60	156.00	156.40	214.00	315.20	339.00
27 April 2009	140.60	146.60	207.20	354.80	435.80	566.40
16 March 2011	111.60	183.00	199.20	287.60	309.00	509.20
3 March 2014	105.60	174.80	181.40	290.60	428.00	738.80
**Thresholds**	**105**	0	0	**193**	0	0

**Table 2:** Evaluation of the performances of the selected thresholds.

	Summary of the Cumulative Events
Events that exceed all the threshold values at the same time	18
Monitored days	6463
Percentage of exceeding thresholds	0.28%
Numbers of cas of exceeding thresholds without event	4
Percentage of case of exceeding thresholds without event	0.06%

## Data Availability

The data are available if required to the authors.
